# Incidence of mental disorders in soldiers deployed to Afghanistan who have or have not experienced a life-threatening military incident—a quasi-experimental cohort study

**DOI:** 10.3389/fpubh.2024.1357836

**Published:** 2024-02-22

**Authors:** Ulrich Wesemann, Karl-Heinz Renner, Katie Rowlands, Kai Köhler, Nils Hüttermann, Hubertus Himmerich

**Affiliations:** ^1^Department of Psychiatry, Psychotherapy and Psychotraumatology, Bundeswehr Hospital Berlin, Berlin, Germany; ^2^Faculty of Human Sciences, Institute of Psychology, Bundeswehr University Munich, Neubiberg, Germany; ^3^Department of Psychological Medicine, King’s College London, Institute of Psychiatry, Psychology and Neuroscience (IoPPN), London, United Kingdom

**Keywords:** military personnel, PTSD, depression, anxiety, mental health, military deployment

## Abstract

**Introduction:**

There is very good international research on deployment-related mental disorders in military personnel. The incidence rates show a very wide range. A new strategy is therefore proposed in order to achieve better standardization and thus better comparability of the studies. In addition to a non-deployed comparison group, we propose to compare deployed soldiers with and without critical military incidents during the deployment. This additional distinction makes it possible to differentiate between the influencing variables of actual threat and general deployment stress.

**Methods:**

*N* = 358 male combat soldiers deployed to Afghanistan were included in the study. Clinical interviews were conducted several days before deployment and after deployment. Of them, *n* = 80 soldiers suffered a life-threatening military incident during deployment, whereas 278 soldiers did not. Odds ratios (OR) were calculated for the groups with and without critical military incidents and the new onset for PTSD, anxiety disorders and depressive disorders.

**Results:**

When comparing both groups, we found significantly higher 1-year incidence rates in the group with critical military incidents: 6.4% vs. 1.1% (OR 6.2) for post-traumatic stress disorder (PTSD); 7.0% vs. 1.1% (OR 6.5) for depression; and 15.9% vs. 2.8% (OR 6.6) for anxiety disorders. The 1-year incidence rate of mental multimorbidity (PTSD with anxiety or depression) was 4.8% vs. 0.4% (OR 12.0).

**Discussion:**

These results indicate that life-threatening military incidents during military deployment are important to mental health. As the different threat levels of the various missions are taken into account, additional predictors could be determined more precisely in further research.

## Introduction

There is an abundance of literature on the prevalence and incidence rates of post-traumatic stress disorder (PTSD) among emergency responders (1–3) and civilians (4, 5) after major disasters. As PTSD is only one mental health outcome among others, common mental disorders such as depression or anxiety are often not recorded. A methodological problem with many studies lies in the naturalistic design. As major disasters are difficult to predict, it is not possible to carry out baseline measurements. The pure prevalence rate of mental disorders can therefore only be attributed to the events to a limited extent. In addition, the informative value is often impaired by the fact that no comparison group is included or available. For example, after the WTC terrorist attacks of 9/11, it is virtually impossible to identify emergency services personnel who were not deployed there in any way after the attack. To address this issue, meta-analyses can help reconcile such differences. However, the problem that often remains is that the clinical and methodological heterogeneity of the studies is very high. This is also significantly limiting the substance of the meta-analyses (6).

For military personnel deployed in war zones, it is somewhat easier, as the missions are more predictable and therefore allow for baseline measurements. Thus, there is a good database on mental disorders after deployments abroad (7–9), while the results of incidence rates are inconsistent in terms of timing and data collection (10, 11). Comparability is also limited by a variety of missions with different military conflict potential as well as different types of data collection, such as the use of questionnaires, clinical interviews or register studies (12, 13). As a result, the incidence rates of deployment-related mental disorders among this population vary widely, e.g., the range for PTSD is between 0.1% (14) and 27.8% (15).

Most studies include undeployed soldiers who refer to comparable units as a control group (16). Differences in the development of mental disorders can therefore be attributed to the deployment. Nevertheless, since the circumstances of deployments abroad differ from those in the home country, this comparison is somewhat problematic. Personal restrictions such as absence from home, climate changes, constant readiness, carrying out operations under different threat conditions, etc. are part of everyday life here. This means that different situations are compared over time, which is the intent of the studies, but each situation is part of the military’s self-image. Although there is no doubt that a deployment abroad increases the burden and stress level, it is only likely to contribute to the development of mental disorders in a few cases. Rather, military threats such as active combat operations, defined as violence by armed forces against people, animals or objects or violence against armed forces through attacks, mines, firefights, etc., are likely to be responsible (17–19).

Following on from these considerations, an extended approach would be to compare soldiers who are on the same deployment during the same period of time. The discrimination could then be made between a risk group of soldiers with critical military incidents, defined as events with a real risk of injury or death, and a comparison group of soldiers without such an event during deployment. The delta of new occurrences of mental disorders could thus be attributed more directly to these war-related events. This would make it easier to compare the different studies and probably lead to more consistent results. As the different threat levels of the various deployments are thus taken into account, other important predictors such as age, gender, rank, duration of deployments, equipment (20), etc. could be determined more precisely. On the other hand, different types of stressors could also be investigated in these missions. Nordstrand and colleagues found that Norwegian soldiers deployed to Afghanistan showed higher posttraumatic deprecation following the non-threat-based stressors “moral challenge or witnessing” than soldiers who experienced “personal threat” (21).

PTSD is one of the most common deployment-related mental disorder in the German Armed Forces (22). A study examining the 1-year incidence rate among German soldiers deployed to Afghanistan found that 2.1% had a new onset of PTSD (unweighted), compared to 0.2% of never-deployed soldiers (23).

To examine the extent to which critical military incidents are responsible for the incidence, we compared military personnel with and without these events. We hypothesized that soldiers exposed to a critical military incident during deployment are more likely to develop PTSD, anxiety and depressive disorders as well as “multimorbidity” defined as PTSD with a depressive or an anxiety disorder.

## Methods

Eligible participants were military personnel of an entire combat troop contingent deployed in Afghanistan from November 2013 to July 2014. These troops were part of the International Security Assistance Force (ISAF) which was a multinational military mission in Afghanistan from 2001 to 2014. The baseline assessment was embedded in the deployment training in November 2013 and took place a few days before the deployment. The second survey was conducted one year after the baseline assessment and had the same framework conditions. From a total of 511, *n* = 496 servicewomen and men (97%) were recorded at the first measurement point. There were *n* = 135 (27%) drop-outs between the two assessments. The main reasons for the dropouts were changes in the military resource planning and medical conditions. We excluded *n* = 3 female soldiers due to lack of representativeness of such a small subgroup, thus *N* = 358 male combat soldiers remained.

Data collection consisted of the clinical interview Mini Diagnostic Interview for Mental Disorders (24) according to ICD-10. Therefore, troop psychologists were trained in this interview. After the training, the agreement of diagnoses between the troop psychologists was 95% (inter-rater reliability). That is acceptable. This interview covers the most common mental disorders, including PTSD, anxiety disorders and depressive disorders.

By comparing the two assessments, it is possible to calculate the new onset of mental disorders after deployment. In the present data analysis, we examined the 1-year incidence rate of mental disorders in *N* = 358 physically uninjured male combat soldiers. The focus was on PTSD, anxiety disorders and depressive disorders. For this purpose, all anxiety disorders, consisting of specific phobias, obsessive-compulsive disorders, agoraphobia (with and without panic attacks), panic attacks, social phobias and generalized anxiety disorders, as well as all depressive disorders, consisting of major depression, dysthymia and hypomania, were summarized.

With respect to the methodological considerations, we compared the incidence rates of 80 combat soldiers who suffered a critical military incident, and 278 who did not. All soldiers of the same rank had the same risk of encountering critical military incidents during deployment resulting in a quasi-experimental design of the study. Sociodemographic characteristics were compared between both groups using *t*-tests for independent samples.

**Figure 1:**
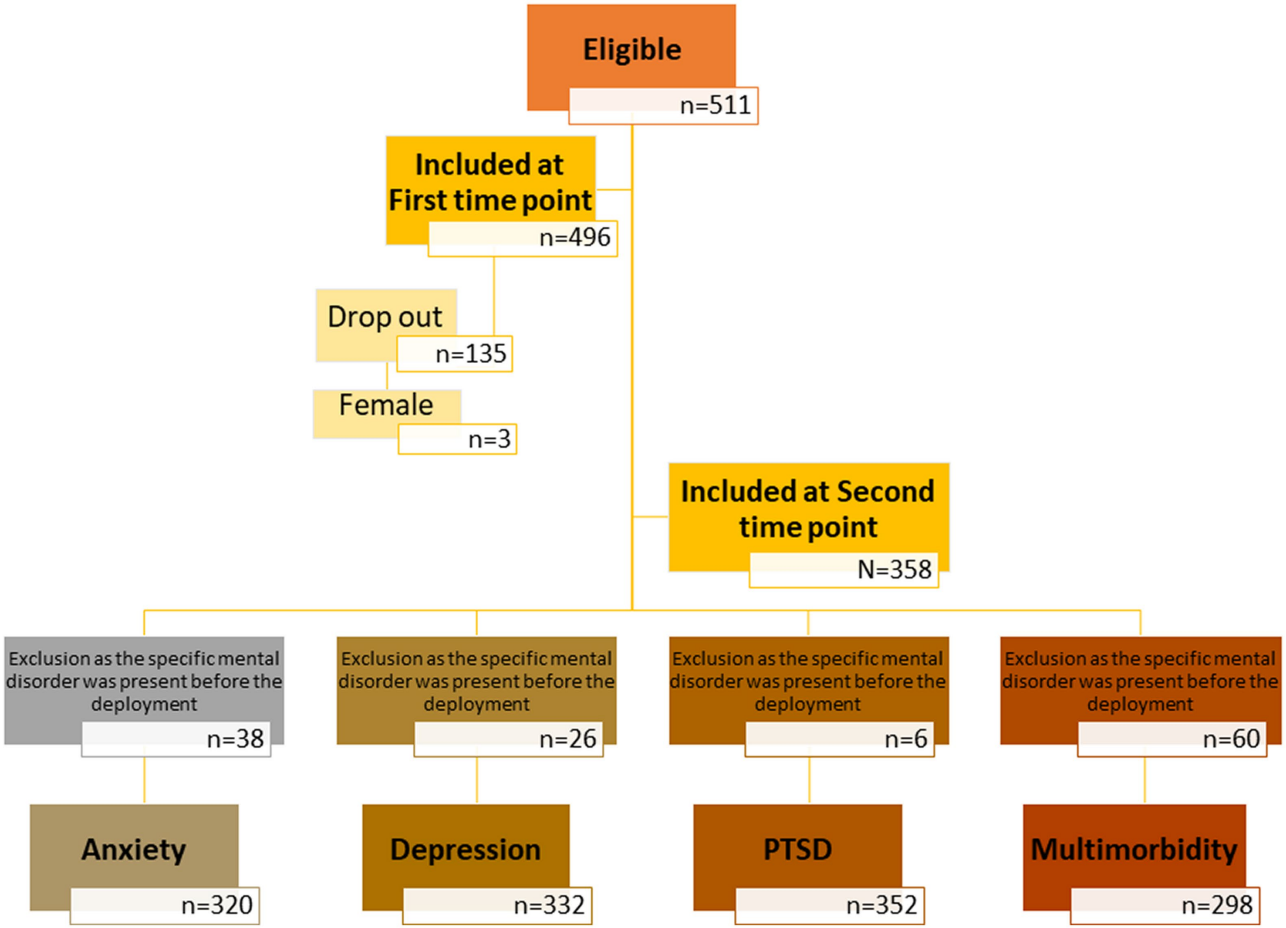
Flowchart illustrating the inclusion of participants in each phase.

Odds ratios (OR) were calculated for the groups with and without critical military incidents sand each mental health outcome for PTSD, anxiety disorders and depressive disorders. Soldiers with the specific pre-deployment diagnosis were each excluded because they were “not at risk” of developing the same diagnosis again (see Figure 1). To test for confounding variables, analysis of covariance (ANCOVA) was performed including the new onset of the specific mental disorders as dependent variable, critical incidents (yes/no) as independent variable and sociodemographic data as covariates.

This cohort study was commissioned by the Federal Ministry of Defence (PIII5-Az-66-55-05). The original research question of the Federal Ministry of Defense related to “Psychological Fitness” and participation was mandatory. Obtaining a written informed consent was not applicable for this project. However, the participants were informed about the meaning and purpose of the study. To comply with the ethics regulations, we therefore obtained approval from the ethics committee of the Bundeswehr University Munich (No: EK UniBw M 23-01) for this research project. In addition, the approval of the Federal Ministry of Defense was obtained for this publication. Further methodological details and legal aspects are published elsewhere (25).

## Results

Age ranged from 18 to 49 years (mean 26.6 years, standard deviation 4.9; median 25 years, interquartile range 23–28 years). Participants were enlisted soldiers (*n* = 211, 58.9%), non-commissioned officers (*n* = 111, 31.0%), or officers *n* = 32 (8.9%). There was a small amount of missing data (*n* = 4; 1.1%).

Comparing both groups, with and without critical military incidents, there were no differences in sociodemographic data as shown in Table 1. The 1-year incidence rates were found for PTSD with 6.4% vs. 1.1%, depression 7.0% vs. 1.1%, and anxiety disorders 15.9% vs. 2.8% with OR ranging from 6.2 to 6.6. The 1-year incidence rate of mental multimorbidity (PTSD with anxiety or depression) was 4.8% vs. 0.4% (OR 12.0). All comparisons revealed statistically significant differences. Table 2 shows the 1-year incidence rates for the subgroups and total group.

**Table 1:** *t*-test for group differences between soldiers with and without critical incidents during deployment in relation to demographic characteristics.

	Deployment	*N*	Mean	SD	*T*-value	df	*p*-value
Age	With critical incident	79	26.5	4.85			
	Without	277	26.6	4.95	−0.09	354	0.933
Rank	With critical incident	78	1.6	0.71			
	Without	276	1.5	0.64	1.30	352	0.209
N_Depl.	With critical incident	80	0.8	1.03			
	Without	278	0.8	1.22	−0.40	356	0.691

SD, standard deviation; *p*-value, significance 2-tailed; N_Depl., number of deployments; YoS, years of service. Differences in df are due to missing data. df with decimals were corrected due to variance heterogeneity using Welch’s test.

**Table 2:** 1-year incidence rates for male German military personnel deployed to Afghanistan with and without critical military incidents.

	Total group *n*/*N* (%) 95% CI	Without CrI *n*/*N* (%) 95% CI	With CrI *n*/*N* (%) 95% CI	OR (w/o CrI) (95% CI)	Χ^2^ (w/o CrI)	*p*
PTSD	8/352 (2.3%) 0.7–3.8	3/274 (1.1%) 0–2.9	5/78 (6.4%) 3.1–9.7	6.19 (1.45–26.50)	7.7	0.005
Depression	8/332 (2.4%) 0.8–4.1	3/261 (1.1%) 0–3.0	5/71 (7.0%) 3.5–10.6	6.52 (1.52–27.96)	8.2	0.004
Anxiety	18/320 (5.1%) 3.1–8.2	7/251 (2.8%) 0–5.6	11/69 (15.9%) 10.6–21.3	6.61 (2.46–17.79)	17.6	<0.001
Multi-morbidity	4/298 (1.3%) 0.0–2.6	1/236 (0.4%) 0–1.9	3/62 (4.8%) 2.0–7.7	11.95 (1.22–116.95)	7.2	0.007

Data are reported as *n* incidence/*n* soldiers at risk (%) 95% CI significance. CrI, critical incident; OR, odds ratio; w/o, with vs without; *p*, level of significance; PTSD, post-traumatic stress disorder; multimorbidity, PTSD with depression or anxiety.

Although participants represent a relatively homogeneous group, ANCOVAs were conducted to test whether other factors influence the higher incidence of these mental disorders among soldiers with critical military incidents. There was no significant effect of covariates on the incidence of depressive disorders, anxiety disorders, or multimorbidity, but critical incidents remained statistically significant. As shown in Table 3, the sociodemographic data had no significant influence on the incidence of PTSD.

**Table 3:** ANCOVA to test the influence of critical incidents during deployment on the incidence of PTSD including number of previous deployments, age, and rank as covariates.

Predictors	*df*	*F*	Sig.	*R* ^2^	*ƞ* ^2^
Number of previous deployments	1	2.10	0.148		0.006
Age	1	0.19	0.667		0.001
Rank	1	0.15	0.695		<0.001
Critical incident	1	8.49	0.004		0.024
Total	4 (348)	3.90	0.004	0.043	0.043

Df, degrees of freedom; *F*, *F*-value; Sig., significance; *R*^2^, coefficient of determination; *ƞ*^2^, partial eta square.

## Discussion

As expected, the group that faced critical military incidents during deployment had significantly higher 1-year incidence rates for all investigated mental disorders. This was particularly pronounced in the comorbidity of PTSD with anxiety or depression. These results indicate that it is more meaningful to compare the outcomes of soldiers who experienced life-threatening military incidents to soldiers on the same deployment who did not experience such an incident, since the living conditions for both groups are comparable. The 1-year incidence for PTSD in our total group with 2.3% corresponds to the unweighted 2.1% 1-year incidence found by Wittchen and colleagues in German soldiers deployed to Afghanistan (23) and to 2.9% each for PTSD and depression found in a representative sample of U.S. Reserve and National Guard (26). The added value is the differentiation of groups with and without critical military incidents during deployment, resulting in an increased 1-year incidence of PTSD with 6.4% for the at-risk group with these incidents. The higher 1-year incidence of PTSD in our sample with no critical military incidents, compared to the never-deployed soldiers (23) of 1.1% vs. 0.2%, could be due to possible differences in exposure to critical events outside the military. In addition, the stressful environment abroad and the reintegration in the home country confer a higher vulnerability to these episodes, supporting our research strategy.

In a recently published study, Dyball and colleagues (27) examined the mental health point prevalence several years after deployment of physically injured and uninjured male military personnel. Soldiers exposed to critical military events during their deployment in Afghanistan up to 2014 were included. Compared to the uninjured group (22), our 1-year incidence rates in the total group (uninjured group with and without critical incidents) are noticeably lower in all categories. Comparing the soldiers with critical military incidents in our study to the uninjured group in the study by Dyball and colleagues (27), the rates are comparable and only remain lower for depression (7.0% vs. 16.8%) in our sample.

In addition to possible differences in the exposure to critical military incidents, the lower depression rate in our sample could be due to several factors. Firstly, results from questionnaires often overestimate mental disorders due to the specificity of the tests. Secondly, we calculated incidence rates and therefore soldiers with previous but ongoing mental disorders are not included. Finally, a large interval between deployment and testing confers a high probability of exposure to additional military and non-military life-critical incidents. We believe that the data from Dyball and colleagues (27) and ours would be more comparable if these factors were considered. This could be achieved in a collaborative project as suggested by Himmerich and colleagues (28).

While our results are not surprising, they can nonetheless be used by policy and stakeholders to address risk factors more specifically, report critical military incidents more clearly, and appreciate these incidents as occupational hazards. Since the conditions for both groups are comparable, the increased OR can most likely be attributed to the critical military incidents. This association has long been recognized for PTSD. However, our data also show such an effect for anxiety disorders and depressive disorders. In the future, this could make it even easier for those affected to have their symptoms recognized as an operational injury and thus as an occupational disease. Screening this risk group for psychological symptoms over a longer period of time seems sensible, as already suggested by Stene et al. (29) and Pirard et al. (30). Our results might also be used for psychoeducational and destigmatization programs for military personnel, as well as previously proposed virtual (31) or other pre- (32) and post-deployment training programs (33–37).

A limitation of our study results is the lack of differentiation between the various critical military incidents during the deployment and the lack of comparison between military ranks. This is due to the relatively small number of soldiers exposed to these incidents, making such sub-samples too small. In addition, the obligatory nature of data collection may have introduced biases in the responses received. It is also known that the perceived threat (38) and the witnessing of acts of violence (39) have a major influence on the psychological consequences. This was not recorded in this study, but should be taken into account in similar studies. This can make it easier to differentiate between the groups of those directly affected, those observed and those not involved. In addition, it would have been helpful to include traumatic experiences prior to deployment in the ANCOVA.

The risk of developing a mental disorder during deployment is significantly higher for members of the armed forces who experience life-threatening military incidents than for those who do not. However, the stress factors of foreign deployments alone, such as the general threat situation, appear to increase the risk. We do not have another control group of non-deployed military personnel from the same time period. However, a comparison with another study (23) shows a higher incidence of PTSD in our deployment group without life-threatening military incidents at 1.1% compared to 0.2%. This leads to the conclusion that other critical events such as sexual assaults, fear of attacks, mines, etc. also play an important role. It is therefore quite possible that both groups also exhibited more severe symptoms, but these were not sufficient for a diagnosis of PTSD, as has already been reported in other studies (40).

## Data availability statement

The data analyzed in this study is subject to the following licenses/restrictions: given the sensitivity of the mandatory study design, the data were not made publicly available. Data requests can be directed to the corresponding author. They are then checked on a case-by-case basis and require the approval of the Federal Ministry of Defence. Requests to access these datasets should be directed to uw@ptzbw.org.

## Ethics statement

The studies involving humans were approved by Bundeswehr University Munich (No: EK UniBw M 23-01). The studies were conducted in accordance with the local legislation and institutional requirements. Written informed consent for participation was not required from the participants or the participants’ legal guardians/next of kin because the original research question of the Federal Ministry of Defense related to “Psychological Fitness” and participation was mandatory.

## Author contributions

UW: Conceptualization, Data curation, Investigation, Methodology, Writing – original draft. K-HR: Methodology, Supervision, Validation, Writing – review & editing. KR: Formal analysis, Supervision, Validation, Writing – review & editing. KK: Formal analysis, Supervision, Validation, Writing – review & editing. NH: Formal analysis, Validation, Writing – review & editing. HH: Conceptualization, Formal analysis, Methodology, Supervision, Validation, Writing – review & editing.
